# Transcriptome changes and cAMP oscillations in an archaeal cell cycle

**DOI:** 10.1186/1471-2121-8-21

**Published:** 2007-06-11

**Authors:** Anke Baumann, Christian Lange, Jörg Soppa

**Affiliations:** 1 Goethe University, Institute for Molecular Biosciences, Biocentre, Max-von-Laue-Str. 9, 60438 Frankfurt, Germany

## Abstract

**Background:**

The cell cycle of all organisms includes mass increase by a factor of two, replication of the genetic material, segregation of the genome to different parts of the cell, and cell division into two daughter cells. It is tightly regulated and typically includes cell cycle-specific oscillations of the levels of transcripts, proteins, protein modifications, and signaling molecules. Until now cell cycle-specific transcriptome changes have been described for four eukaryotic species ranging from yeast to human, but only for two prokaryotic species. Similarly, oscillations of small signaling molecules have been identified in very few eukaryotic species, but not in any prokaryote.

**Results:**

A synchronization procedure for the archaeon *Halobacterium salinarum *was optimized, so that nearly 100% of all cells divide in a time interval that is 1/4^th ^of the generation time of exponentially growing cells. The method was used to characterize cell cycle-dependent transcriptome changes using a genome-wide DNA microarray. The transcript levels of 87 genes were found to be cell cycle-regulated, corresponding to 3% of all genes. They could be clustered into seven groups with different transcript level profiles. Cluster-specific sequence motifs were detected around the start of the genes that are predicted to be involved in cell cycle-specific transcriptional regulation. Notably, many cell cycle genes that have oscillating transcript levels in eukaryotes are not regulated on the transcriptional level in *H. salinarum*.

Synchronized cultures were also used to identify putative small signaling molecules. *H. salinarum *was found to contain a basal cAMP concentration of 200 μM, considerably higher than that of yeast. The cAMP concentration is shortly induced directly prior to and after cell division, and thus cAMP probably is an important signal for cell cycle progression.

**Conclusion:**

The analysis of cell cycle-specific transcriptome changes of *H. salinarum *allowed to identify a strategy of transcript level regulation that is different from all previously characterized species. The transcript levels of only 3% of all genes are regulated, a fraction that is considerably lower than has been reported for four eukaryotic species (6% – 28%) and for the bacterium *C. crescentus *(19%).

It was shown that cAMP is present in significant concentrations in an archaeon, and the phylogenetic profile of the adenylate cyclase indicates that this signaling molecule is widely distributed in archaea. The occurrence of cell cycle-dependent oscillations of the cAMP concentration in an archaeon and in several eukaryotic species indicates that cAMP level changes might be a phylogenetically old signal for cell cycle progression.

## Background

The cell cycle is characterized by periodic events that have to occur in the lifetime of nearly every cell, e.g. mass increase by a factor of two, DNA replication, DNA segregation, and cell division. The eukaryotic cell cycle includes a stage of high chromosome condensation, resulting in mitotic chromosomes that are visible in the light microscope, and has therefore attracted attention during the last centuries. Interest in the prokaryotic cell cycle has increased substantially during the last decade. Examples for keynote discoveries are: 1) the bacterial chromosome is not randomly distributed in the cell, but is highly organized, 2) replication takes place at midcell at a fixed replisome, while the DNA is actively transported in archaea and bacteria, and 3) specific degradation of cell cycle regulatory proteins occurs at least in bacteria. Several reviews illustrate the state of the art and current questions of cell cycle research with eukaryotes, bacteria, and archaea [1-9]. It should be noted that the research concentrates on very few model species, including 1) the eukaryotes *Saccharomyces cerevisiae, Schizosaccharomyces pombe*, and human cell lines, 2) the bacteria *Caulobacter crescentus, Bacillus subtilis *and *Escherichia coli*, and 3) the archaea *Sulfolobus acidocaldarius *and *Halobacterium salinarum*.

In all three domains of life it was found that the levels of specific transcripts and proteins vary in a cell cycle-dependent manner. The first global analyses of cell cycle-dependent transcript level changes were performed with the budding yeast *S. cerevisiae*, and several hundreds of transcripts were found to oscillate [10,11]. Recently three independent transcriptome studies of the *S. pombe *cell cycle have been reported, and the transcript levels of 400 and 750 genes were found to be cell cycle-regulated [12-14]. A meta-analysis of the three datasets came to the conclusion that the combined dataset allows to identify about 500 genes as being cell cycle-regulated [15]. About the same number of genes were found to be cell cycle-regulated in an *Arabidopsis *cell line. However, the real number in *Arabidopsis *is higher, because a microarray covering only one-third of the genome was used [16]. Until now three independent approaches have been reported that used whole genome DNA microarrays to study transcript level changes within the human cell cycle [17-19]. It was found that 700 and 900 genes are cell cycle regulated in fibroblasts and in HeLa cells, respectively.

In contrast to the availability of 10 independent transcriptome studies with eukaryotes, a single study has been reported for a bacterial species, i.e. *C. crescentus *[20]. It was revealed that the transcript levels of 553 genes are cell cycle-regulated, corresponding to 19% of the genome. Very recently characterization of cell cycle-dependent transcriptome changes have been reported for *Sulfolobus acidocaldarius*, which belongs to the kingdom of Crenarchaeota [21].

A prerequisite for the determination of cell cycle-dependent transcriptome changes is a method to synchronize cell cultures. Cell synchronization procedures have been described for only a very limited number of species. These include budding yeast and fission yeast, human cell lines, *E. coli*, *B. subtilis*, *C. crescentus*, *S. acidocaldarius *and *H. salinarum*.

Here, we present an optimized synchronization procedure for *H. salinarum *that yields a very high degree of synchronization. Synchronized cultures were used to characterize cell cycle-dependent transcriptome changes in an archaeal species. The results are discussed and compared with the results obtained for four eukaryotic and two prokaryotic species. Furthermore, it was revealed that the concentration of the signaling molecule cAMP oscillates within the cell cycle. The results together with the results of an earlier study, e.g. the characterization of cell cycle-dependent dynamic intracellular localization of the DNA, yield an integrated overview of an archaeal cell cycle.

## Results

### Synchronization of *H. salinarum *cultures

**Figure 1:**
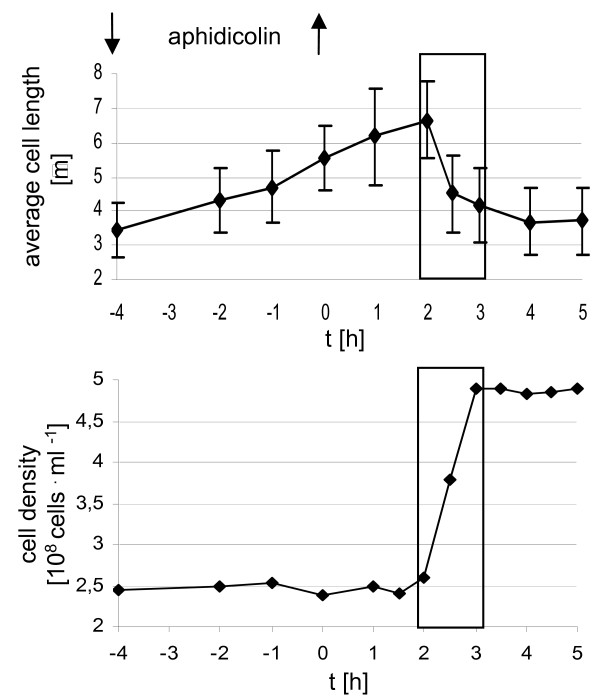
**Optimized synchronization of *H. salinarum *cultures**. The synchronization was performed as described in Experimental Procedures using the DNA polymerase inhibitor aphidicolin. The average cell length and its standard deviation was calculated from the lengths of 50 cells that were determined microscopically with an ocular micrometer. The cell density was determined microscopically with a Neubauer counting chamber. Times of addition and removal of the inhibitor are indicated. The time of inhibitor removal was set to zero to allow a direct comparison of the times shown in this and additional Figures (2 – 6, see Additional file 1). The box in this and additional Figures denotes the only time interval in which dividing cells with visible constrictions could be observed. Microscopic images of dividing cells and the intracellular DNA localization have been published previously [22].

The previously described synchronization procedure for *H. salinarum *involves treatment of an exponentially growing culture with the DNA polymerase inhibitor aphidicolin for four hours. After inhibitor removal and cell washing, a synchronized culture was obtained [22]. Different steps of the procedure were optimized. The most important points are 1) that the number of generations of exponential growth prior to synchronization was considerably increased (now growth for more than 40 generations is routinely used), and 2) that the culture is not treated with aphidicolin for a fixed time span, but aphidicolin is removed as the cells reach an average length of 5.7 μm. Fig. 1 shows the cell density and the average cell length profiles of a culture treated according to the optimized protocol. Nearly 100% of all cells divide within one hour, which is short in comparison to the four hour generation time of exponentially growing *H. salinarum*. Cells with a visible constriction were seen exclusively during this one hour period (Fig. 1). The high degree of synchrony makes *H. salinarum *cultures optimally suited to study cell cycle-related processes, e.g. differential transcript level regulation.

To address the question of how long synchrony persists, we characterized a culture for two cell cycles following synchronization. As it is typical for whole culture synchronization methods, it turned out that the degree of synchrony is higher for the first than for the second cycle and thus the first cycle is more informative (data not shown). Therefore we chose to concentrate on the first cycle for the characterization of cell cycle-dependent transcriptome changes, which allowed to analyze the transcriptome at a high number of time points.

### Cell cycle-dependent transcriptome changes

For the characterization of cell cycle-dependent transcriptome changes a DNA microarray was used that had been constructed in collaboration with the group of Dieter Oesterhelt (MPI for Biochemistry, Martinsried, Germany). It is comprised of gene-specific PCR products that represent more than 95% of all genes that were annotated in the course of the *H. salinarum *genome project [23].

Aliquots of a synchronized culture were removed at 10 time points and used for RNA isolation. As a control, a culture was used that was treated identically except that the aphidicolin addition was omitted. Generation and labeling of cDNAs, competitive hybridization, DNA microarray processing and data handling are described in the Methods section. Three independent experiments (biological replicates) were performed and the results were analyzed as described (Methods). The number of genes with cell cycle-regulated transcript levels is surprisingly small. Out of 2457 genes that generated signals for at least seven time points, only the transcript levels of 87 genes were found to be cell cycle-regulated. These genes are summarized in Table 1. They are not randomly distributed in the genome, but more than half of them were found to be part of 16 gene clusters or operons (Table 1). 22 genes are annotated as "hypothetical" or "conserved hypothetical", but the characterization of their cell cycle-regulated expression has proven that these are real genes.

**Table 1:** Genes with cell cycle-specific transcripts

**No.**	cluster*^1^	identifier*^2^	g.c./ow*^3^	**gene name**	**functional class**
1	1	OE2697R		probable coenzyme PQQ synthesis protein E	coenzyme metabolism
2	1	OE3763F	I	glycerol-3-phosphate dehydrogenase chain A	central intermediary metabolism
3	1	OE3764F	I	glycerol-3-phosphate dehydrogenasechain B	central intermediary metabolism
4	1	OE3765F	I	glycerol-3-phosphate dehydrogenase chain C	central intermediary metabolism
5	1	OE4345R		probable ribonucleoside-diphosphate reductase	nucleotide metabolism
6	2	OE1806R		probable periplasmic protein	cell envelope
7	2	OE2844R		transcription regulator homolog/*trkA *C-terminal domain	gene regulation
8	2	OE3308F		malate dehydrogenase	central intermediary metabolism
9	2	OE3759R		hypothetical protein	hypothetical protein
10	2	OE3922R		glutamate-ammonia ligase (EC 6.3.1.2)	amino acid metabolism
11	2	OE5212F		SMC-like protein *sph1*	cellular processes
12	3	OE1620R		phosphoribosylglycinamide formyltransferase	nucleotide metabolism
13	3	OE1951F	I	phosphoribosylaminoimidazole carboxylase (EC 4.1.1.21)	nucleotide metabolism
14	3	OE1952F	I	phosphoribosylaminoimidazole carboxylase (EC 4.1.1.21)	nucleotide metabolism
15	3	OE2274R		phosphoribosylformylglycinamidine synthase (EC 6.3.5.3)	nucleotide metabolism
16	3	OE2864F		phosphoribosylamine--glycine ligase (EC 6.3.4.13)	nucleotide metabolism
17	3	OE3017R		UDP-sugar hydrolase (EC 3.6.1.45)/5'-nucleotidase	nucleotide metabolism
18	3	OE3139R		amidophosphoribosyltransferase (EC 2.4.2.14)	nucleotide metabolism
19	3	OE3724F		phosphoribosylaminoimidazolesuccinocarboxamide	nucleotide metabolism
				synthase	
20	4	OE1500R		pyruvate, water dikinase (EC 2.7.9.2) (PEP synthase)	central intermediary metabolism
21	4	OE2019F		fructose-bisphosphate aldolase (EC 4.1.2.13) 1	central intermediary metabolism
22	4	OE4416R	I	conserved protein	conserved hypothetical protein
23	4	OE4418R	I	hypothetical protein	hypothetical protein
24	4	OE4419R	I	argininosuccinate lyase (EC 4.3.2.1)	amino acid metabolism
25	4	OE6026R		transcription initiation factor TFB	transcription
26	5	OE1058R		probable transposase (ISH1)	transposases/ISH proteins
27	5	OE1270F	I	glutamate dehydrogenase (EC 1.4.1.-)	amino acid metabolism
28	5	OE1271F	I	probable fatty-acid--CoA ligase (EC 6.2.1.-)	lipid metabolism
29	5	OE1352F	I	hypothetical protein	hypothetical protein
30	5	OE1353F	I	hypothetical protein	hypothetical protein
31	5	OE1356F	I	conserved hypothetical protein	conserved hypothetical protein
32	5	OE1447R	I	conserved hypothetical protein	conserved hypothetical protein
33	5	OE1448R	I	conserved hypothetical protein	conserved hypothetical protein
34	5	OE1775R		hypothetical protein	hypothetical protein
35	5	OE2046F		conserved hypothetical protein	conserved hypothetical protein
36	5	OE2492F		conserved protein	conserved hypothetical protein
37	5	OE3168R		pyridoxal phosphate-dependent aminotransferase	miscellaneous
38	5	OE3554F		carbamoyl-phosphate synthase	amino acid metabolism
39	5	OE3612R		chemotactic signal transduction system	signal transduction
				periplasmic substrate-binding protein *basB*	
40	5	OE3653R	I	phosphoglycerate mutase (EC 5.4.2.1)	central intermediary metabolism
41	5	OE3654R	I	hypothetical protein	hypothetical protein
42	5	OE3925R		thermosome beta chain	cellular processes
43	5	OE4122R		thermosome alpha chain	cellular processes
44	5	OE4159F		adenosylhomocysteinase (EC 3.3.1.1)	amino acid metabolism
45	5	OE4300R	I	hypothetical protein	hypothetical protein
46	5	OE4301R	I	ABC-type transport system ATP-binding protein	small molecule transport
47	5	OE4302R	I	ABC-type transport system ATP-binding protein	small molecule transport
48	5	OE4303R	I	ABC-type transport system permease protein	small molecule transport
49	5	OE4304R	I	ABC-type transport system permease protein	small molecule transport
50	5	OE4311F	I	ABC-type transport system substrate-binding protein	small molecule transport
51	5	OE4316F	I	ABC-type transport system permease protein	small molecule transport
52	5	OE4318F	I	ABC-type transport system ATP-binding protein	small molecule transport
53	5	OE4408F	I	phosphoglycerate dehydrogenase (EC 1.1.1.95)	amino acid metabolism
54	5	OE4410F	I	acyl-CoA thioester hydrolase homolog	conserved hypothetical protein
55	5	OE4550F	I	ABC-type transport system ATP-binding protein	small molecule transport
56	5	OE4552F	I	ABC-type transport system permease protein	small molecule transport
57	5	OE4555F	I	ABC-type transport system permease protein	small molecule transport
58	5	OE4676F		hypothetical protein	hypothetical protein
59	5	OE4688F		conserved protein	conserved hypothetical protein
60	6	OE1794R	I	conserved hypothetical protein	conserved hypothetical protein
61	6	OE1797R	I	transcription regulator *sirR*	gene regulation
62	7	OE1674R	I	probable phosphate transport operon protein *phoU*	transport
63	7	OE1675R	I	ABC-type transport system ATP-binding protein	small molecule transport
64	7	OE1676R	I	ABC-type transport system permease protein	small molecule transport
65	7	OE1678R	I	ABC-type transport system permease protein	small molecule transport
66	7	OE1679R	I	ABC-type transport system periplasmic binding protein	small molecule transport
67	7	OE2367F		aldehyde dehydrogenase	miscellaneous
68	7	OE2458R		IMP dehydrogenase (EC 1.1.1.205)	nucleotide metabolism
69	7	OE2924R		conserved hypothetical protein	conserved hypothetical protein
70	7	OE3571R	I	GMP synthase (glutamine-hydrolyzing) (EC 6.3.5.2)	nucleotide metabolism
71	7	OE3572R	I	CTP synthase (EC 6.3.4.2)	nucleotide metabolism
72	7	OE4462F		conserved hypothetical protein	conserved hypothetical protein
73	7	OE4466R		DNA repair protein	replication, repair, recombination
74	7	OE5160F		glycerol dehydrogenase (EC 1.1.1.6)	miscellaneous
75	7	OE5204R	I	arginine ornithine exchanger	small molecule transport
76	7	OE5205R	I	ornithine carbamoyltransferase (EC 2.1.3.3)	amino acid metabolism
77	7	OE5206R	I	carbamate kinase (EC 2.7.2.2)	amino acid metabolism
78	7	OE5208R	I	arginine deiminase (EC 3.5.3.6)	amino acid metabolism
79		OE1249R		conserved hypothetical protein	conserved hypothetical protein
80		OE1302F		lipoate-protein ligase homolog	miscellaneous
81		OE2703F		probable copper-containing oxidoreductase	miscellaneous
82		OE2973F		conserved hypothetical protein	conserved hypothetical protein
83		OE3337F		insertion element protein (ISH2)	transposases/ISH proteins
84		OE4077F		hypothetical protein	hypothetical protein
85		OE4380F		cell division control protein *cdc6 *homolog	cellular processes
86		OE7065F	I	cytochrome d ubiquinol oxidase (EC 1.10.3.-) chain I	energy metabolism
87		OE7066F	I	cytochrome d ubiquinol oxidase (EC 1.10.3.-) chain II	energy metabolism

**Figure 2:**
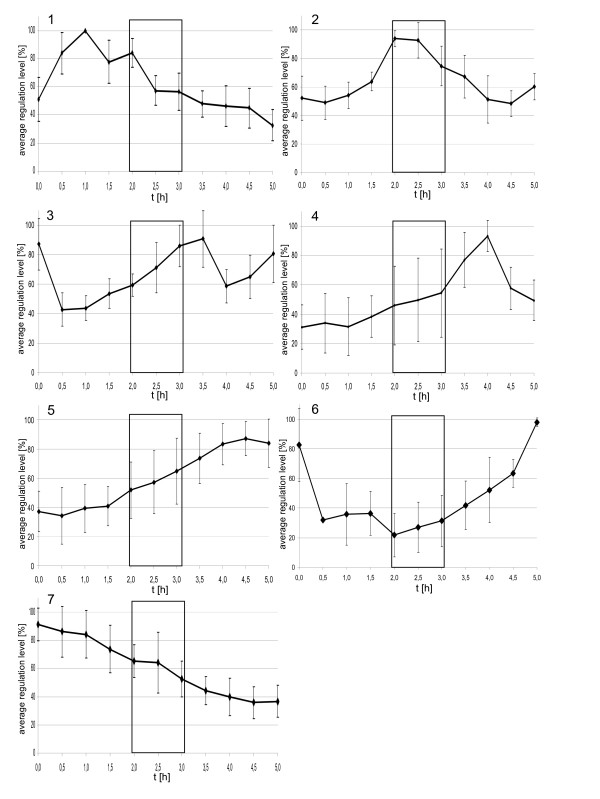
**Average transcript level profiles of seven clusters of co-regulated genes**. Most of the cell cycle-regulated transcripts were sorted into seven clusters of co-regulated genes (compare text and Table 1). The average transcription profiles of all seven clusters and the standard deviations are shown. Genes that share an identical profile of induction and repression do not necessarily share the same degree of induction/repression, therefore the transcript profiles of all genes were normalized to their highest value (= 100%) before calculation of averages and standard deviations. Gene identifiers, names, and functional classes are summarized in Table 1.

*^1 ^regulatory cluster (compare Fig. 2)

*^2 ^identifier in the genome sequence [23]

*^3 ^gene cluster or operon

The 87 genes were grouped, based on their cell cycle-specific transcriptional profiles, and seven clusters of co-regulated genes were discovered. The average transcript level profiles of all seven clusters are shown in Fig. 2. Clusters one to four are comprised of transcripts that were transiently up-regulated in the course of the cell cycle and relax back to their original level (25 genes). The time of maximal transient induction is different for the four clusters, e.g. one hour after inhibitor removal for cluster one and three hours later for cluster four. Cluster five is characterized by transcripts that have a constant transcript level for the first 1.5 h after inhibitor removal and are induced thereafter (34 genes). Cluster six contains transcripts that were transiently down-regulated (2 genes), with a minimal transcript level at the time of cell division. Cluster seven contains transcripts that were down-regulated in the five hours after inhibitor removal (17 genes). The expression profiles of only nine genes did not belong to any of the seven clusters (Table 1).

### Verification of dynamic transcript level patterns

**Figure 3:**
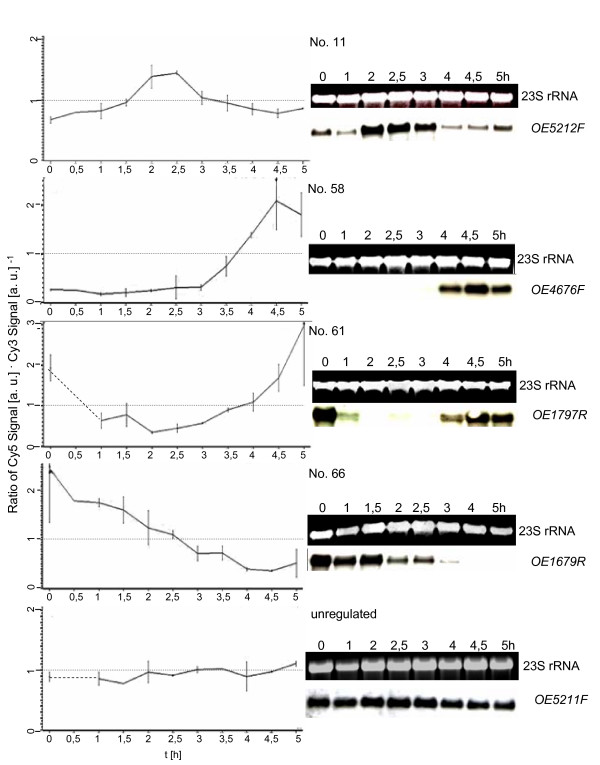
**Comparison of results obtained by DNA microarray analysis and by Northern blot analysis**. 13 genes were selected for verification of the microarray results with an independent method, i.e. Northern blot analysis. They represent all clusters of co-regulated genes as well as unregulated control genes. The transcript level profiles obtained for individual genes by microarray analysis are shown on the left side (average of three biological replicates). On the right side the results of Northern blot analysis are shown (one typical experiment). Gene identifier [23] and the gene No. in Table 1 are included.

**Figure 4:**
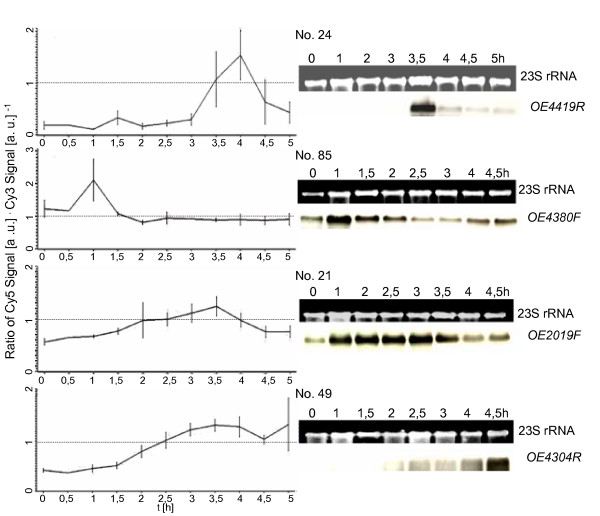
**Comparison of results obtained by DNA microarray analysis and by Northern blot analysis**. 13 genes were selected for verification of the microarray results with an independent method, i.e. Northern blot analysis. They represent all clusters of co-regulated genes as well as unregulated control genes. The transcript level profiles obtained for individual genes by microarray analysis are shown on the left side (average of three biological replicates). On the right side the results of Northern blot analysis are shown (one typical experiment). Gene identifier [23] and the gene No. in Table 1 are included.

Ten cell cycle-regulated genes representing all different transcriptional profiles and three unregulated control genes were chosen arbitrarily and used for verification of the DNA microarray results with an independent method. Aliquots were removed from synchronized cultures at eight time points and used for RNA isolation. The amount of transcript of the 13 genes was determined by Northern blot analyses with gene-specific probes. Fig. 3, 4, 5 shows a comparison of the results of the DNA microarray analysis (average of three biological replicates) and of the Northern blot analysis (one typical experiment). In all cases the results generated by the two different methods are in congruence, underscoring the quality of the microarray data.

**Figure 5:**
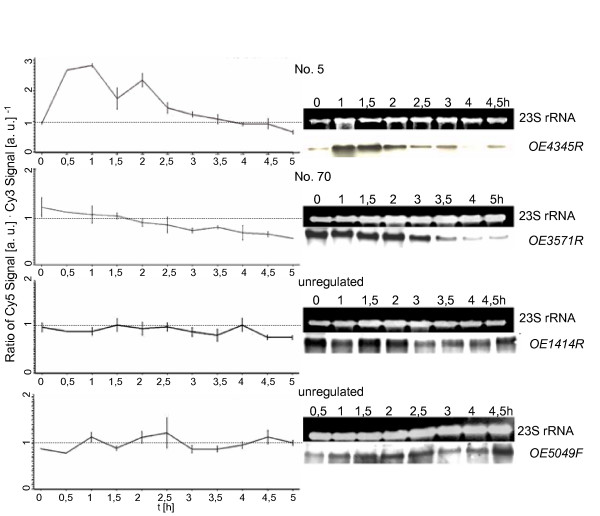
**Comparison of results obtained by DNA microarray analysis and by Northern blot analysis**. 13 genes were selected for verification of the microarray results with an independent method, i.e. Northern blot analysis. They represent all clusters of co-regulated genes as well as unregulated control genes. The transcript level profiles obtained for individual genes by microarray analysis are shown on the left side (average of three biological replicates). On the right side the results of Northern blot analysis are shown (one typical experiment). Gene identifier [23] and the gene No. in Table 1 are included.

### Cell cycle-regulated genes of *H. salinarum*

The genes included in the seven clusters with different transcriptional profiles were examined to identify biological processes that are cell cycle regulated in haloarchaea (Table 1 and Fig. 2). Cluster three is comprised exclusively of genes encoding enzymes of the purine biosynthesis pathway (Fig. 2). Of the remaining genes of the pathway, one is not represented on the microarray and one was found to have a nearly constant transcript level. An oscillation of the purine biosynthetic pathway in the cell cycle and the highest level of induction after cell division, prior to the next replication phase, is in line with the biological function. Intuitively, the regulation of the purine genes would be expected to be accompanied by a similar regulation of the pyrimidine biosynthetic pathway. However, none of the genes for pyrimidine biosynthesis are cell cycle-regulated (see Additional file 1), indicating that pyrimidine biosynthesis is regulated differently.

Cluster five contains 34 genes with highest transcript levels about 1.5 hours after cell division (Fig. 2). Several of them had a very low transcript level until cell division was completed and were rapidly induced thereafter, similar to the M/G1 induced genes found in eukaryotes (an example is OE4676F, No. 58 shown in Fig. 3, 4, 5). This cluster is characterized by a very high fraction of genes of unknown function (13 of 34) in comparison to the other clusters. 10 genes encode subunits of three different ABC transporters. These transporters might be specific for substrates that are needed specifically after cell division, e.g. for purines and pyrimidines needed for replication.

Cluster six is comprised of only two genes that form an operon. The two genes overlap by three nucleotides and were proven to be co-transcribed by Northern blot analysis. The transcript levels are down-regulated until the cells divide, and are up-regulated thereafter (Fig. 2). One of the genes encodes a transcriptional regulator (OE1797R). It belongs to COG1321 ("Mn-containing transcriptional regulators") and has orthologs in all archaeal genomes. The second deduced protein (OE1794R) has no similarity to any protein of known function, but is proposed to have five membrane-spanning helices and thus could be a membrane-bound sensor. These gene products might turn out to play an important role for cell cycle progression and will be further characterized.

Cluster seven contains 17 genes whose transcript levels constantly decrease throughout the experiment and are not up-regulated again. Five genes encode an ABC transporter that is annotated to be phosphate-specific, and four genes encode all enzymes for arginine fermentation. The highest level of induction at the time of inhibitor removal and the noncyclic profile make it possible that cluster seven genes were artificially induced due to the synchronization protocol, in spite of the fact that the control culture was treated identically except for aphidicolin addition. However, if this would be true, it would only strengthen the result that the fraction of cell cycle-regulated genes is much lower in *H. salinarum *than in the six other species investigated thus far.

It is noteworthy that the transcript levels of only very few cell cycle genes are cell cycle-regulated. One example is Sph1 (cluster two; No. 11 in Fig. 3, 4, 5), a member of the SMC protein family [24]. It was previously reported to be cell cycle-regulated on the transcript level as well as on the protein level [22]. Another example is a gene encoding a homologue of the eukaryotic protein Cdc6 that is involved in replication initiation (Table 1; No. 85 in Fig. 3, 4, 5). However, the *H. salinarum *genome contains nine *cdc6 *genes, and the other eight genes are expressed constitutively (see Additional file 1).

Transcripts of many more cell cycle genes are cell cycle-regulated in other species (see Discussion). While the regulation is not well conserved, some genes are cell cycle-regulated in two Yeasts, *Arabidopsis*, and humans. To ensure that the regulation of these genes has not escaped the analysis of the microarray results, the transcriptional profiles of 26 genes were checked, encoding i.e. the haloarchaeal histone, subunits of the DNA polymerase, three proteins of the SMC family, several Cdc48 homologs, and several homologs of the cell division control protein Cdc6 (see Additional file 1). Clearly, none of the genes is cell cycle-regulated in *H. salinarum*.

### Detection of conserved cluster-specific DNA motifs

**Figure 6:**
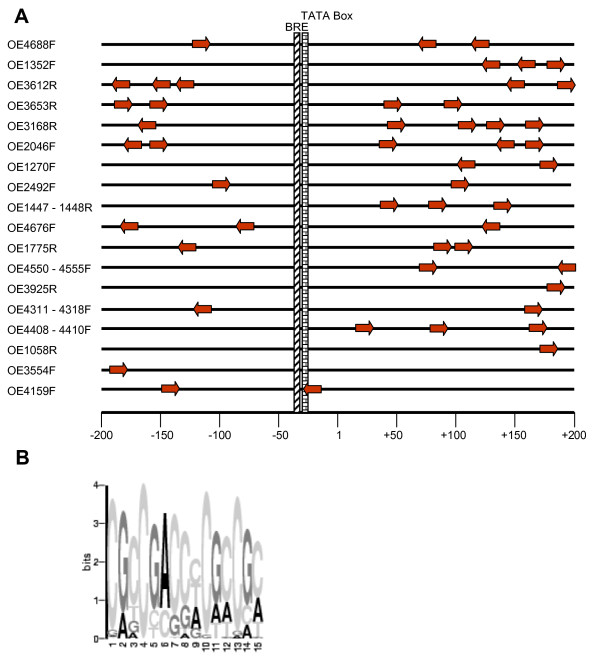
**The conserved motif around the start site of cluster five genes**. The program MEME was used to identify a conserved motif in the 400 nt region around the translational start point of cluster five genes (compare Table 2). **A. **The region is shown schematically, and the numbering refers to the translational start point. Because the majority of haloarchaeal transcripts are leaderless, the transcriptional and the translational start points often nearly coincide. The basal promoter elements "transcription factor B recognition element" (BRE) and "TATA box" are indicated at positions they would have for leaderless transcripts. Gene identifiers [23] are shown to the left. The conserved motif and its direction are indicated by arrows which are drawn to scale. **B. **The sequence logo of the conserved motif of cluster five genes (generated with MEME).

Similar transcriptional profiles of genes indicate shared transcriptional regulators. To identify putative regulatory motifs, cluster-specific multiple sequence alignments were produced including the region from -200 nt to +200 nt centered around the translational start point. This will more or less also align the transcriptional start points, because the majority of haloarchaeal transcripts is leaderless (Brenneis, Hering and Soppa, in preparation). In all cases where the intergenic region between two genes was smaller than 40 nt, they were considered to be part of an operon and only the first gene was included into the analysis. As many genes are part of putative operons (Table 1), the number of cell cycle-specific promoters is considerably smaller than the number of cell cycle-specific genes. Nevertheless, conserved sequence motifs were found for six of the seven clusters. The only exception is cluster six, which is comprised of two genes in one operon and thus could not be analyzed. Table 2 summarizes the consensus sequences, the number of promoters, the average occurrence per promoter, the strand-specificity and the average P-value. Fig. 6A gives an overview of the distribution of the consensus motif around the start site of cluster five genes. Unexpectedly, the motif is found both upstream and downstream of the start site. With only two exceptions the region between -70 nt and +40 nt is totally devoid of the motif. This eliminates the mechanism of transcriptional regulation which was found to be true for most archaeal promoters that have been studied until now, i.e. that binding of a negative regulator inhibits binding of the basal transcription factors TBP and TFB and/or of the RNA polymerase [25]. A sequence logo of the cluster five motif is shown in Fig. 6B. It is extremely GC-rich and includes a short direct repeat, i.e. "CGCCG". Further studies will clarify whether regulatory proteins bind to this and the other cluster-specific consensus sequences and whether they are essential for cell cycle-specific expression of the genes.

**Table 2:** Predicted cis-acting regulatory sequences of cell cycle-regulated gene clusters

cluster	No. promoters	consensus sequence	No. promoters with motif	average No.of occurance per promoter	∅ P-value	strand +/-
1	3	**AMRRCRAC**	3	5	1,60E-04	10/5
1	3	**WGSAGKGSAACCTC**	3	1	2,79E-08	3/0
2	6	**MCGARKWMRAC**	5	2	1,08E-05	7/2
3	7	**THTHCWYKRWSGTGK**	6	2	1,20E-06	3/7
4	4	**SWWCGASSMSG**	4	5	1,60E-04	14/6
5	20	**CRBCGASSDCRHCGH**	18	3	7,88E-05	31/19
6	1	---------	----	----	----	----
7	10	**RHSRYSRMCGA**	10	5	4,16E-04	35/15

R = G or A; Y = T or C; M = A or C; K = G or T; S = G or C; W = A or T; H = A or C or T; B = G or T or C; D = G or A or T

### Cell cycle-dependent levels of the putative signaling molecule cAMP

**Figure 7:**
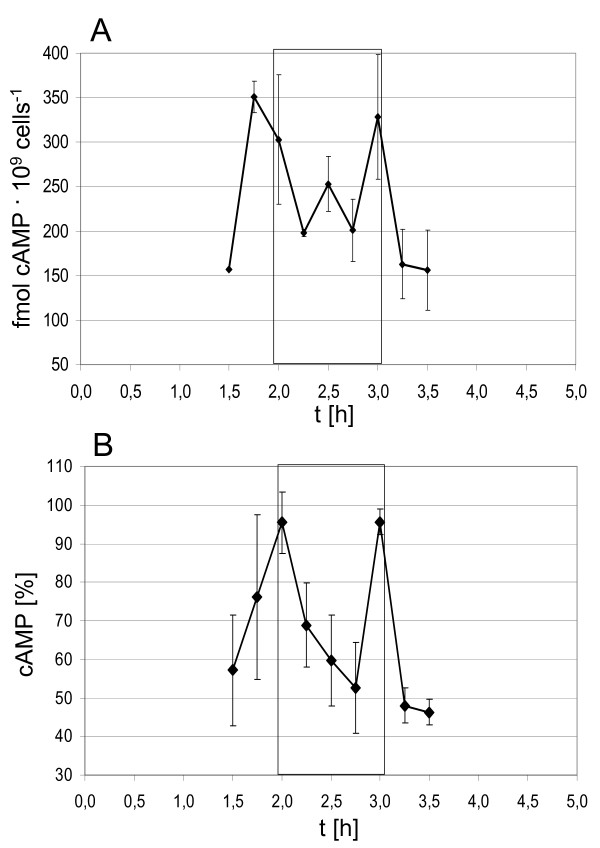
**Cell cycle-dependent changes of the cAMP level**. The cAMP level was determined in synchronized cultures, and it was revealed that concentration changes might occur in the time period between 1.5 and 3.5 hours (data not shown). Therefore this period was characterized with a higher time resolution of 15 minutes. The box in A and B denotes the time period of cell division (compare Fig. 1). **A. **One out of three biological replicates is shown. The cAMP concentration was determined in duplicate measurements, and the cell density was determined with a Neubauer counting chamber. **B. **Average cAMP level changes in three biological replicates. The absolute cAMP values (fmol cAMP/10^9 ^cells) had a somewhat large variance, therefore the highest value in every independent experiment was set to 100% and the relative values were used to calculate the average and the standard deviation.

The small number of cell cycle-regulated genes and the absence of regulation for many genes with conserved cell cycle-specific regulation in eukaryotes indicates that cell cycle-regulation of many *H. salinarum *genes might occur at the posttranscriptional level. Characterization of cell cycle-dependent changes of the proteome will be performed in a subsequent study. Here we report the identification of cell cycle-dependent concentration changes of a small signaling molecule, i.e. cAMP. cAMP was chosen because the cAMP level was found to oscillate in the cell cycle of several eukaryotic species. Haloarchaea are exceptionally well suited for the determination of short-lived metabolites because cell lysis is very rapid after an osmotic downshift. A method for quantitative cAMP determination was established, and it was found that *H. salinarum *cultures growing exponentially in complex medium (2 – 5 × 10^8 ^cells/ml) contain between 200 and 300 fmoles/10^9 ^cells. This is equivalent to about 1 – 2 × 10^5 ^cAMP molecules per cell. Well-energized cultures contain about 5 × 10^6 ^ATP molecules per cell, therefore cAMP is generated from several percent of the available ATP (data not shown). Next, aliquots were removed from synchronized cultures at 30 minutes intervals and the cAMP levels were determined. Three independent experiments did not allow to clarify whether the cAMP levels changed in a cell cycle-dependent manner. It was obvious that no change occurred during the first 1.5 hours and the last 1.5 hours, but the time from 1.5 to 3.5 hours gave inconclusive results (data not shown). Therefore the time resolution was increased and the period from 1.5 to 3.5 hours after aphidicolin removel was investigated in 15 minutes intervals. Fig. 7A shows the result of one out of three independent experiments, and Fig. 7B shows the average cAMP levels of all three biological replicates. The cAMP level rapidly and reversibly increases at two time points just prior to and just after cell division, while it drops to basal level in between. The cell cycle-dependent concentration change of this signaling molecule indicates that it is involved in the regulation of cell cycle processes.

The genome of *H. salinarum *contains a gene for an adenylate cyclase (OE2856F), while similarity searches did not lead to the identification of a phosphodiesterase. The transcript of OE2856F is not cell-cycle regulated, and therefore the cAMP level is regulated post-transcriptionally.

## Discussion

### Synchronization of *H. salinarum *and eukaryotes with aphidicolin block/release protocols

The optimized synchronization procedure yields cultures with a very high degree of synchrony, i.e. nearly 100% of all cells divide in a time period that is equal to 1/4^th ^of the generation time of exponentially growing cells (4 hours). Because the transcript level profiles of the first and the last hour of the five hour period of transcriptome characterization are not identical (Fig. 2 and 3) the first cycle after inhibitor removal seems to be longer than four hours, and thus the cells divide in a fraction of the cell cycle that is even smaller than 1/4. The synchronization method is based on the inhibition of replication by aphidicolin and its subsequent release. A variety of cell cycle features were observed to be synchronized as a result of the block/release protocol of replication: 1) septum formation and cell division, 2) DNA transport and the dynamic intracellular DNA localization [22], 3) transcript levels of seven clusters of genes with different transcript level profiles, 4) the Sph1 protein level [22], and 5) the cAMP concentration. This shows that key elements of the cell cycle are dependent on the successful termination of DNA replication, and a cell cycle checkpoint exists which inhibits cell division in case of replication problems. Importantly, the energy and the anabolic metabolisms are not coupled to replication, and cells elongate with the normal rate of unsynchronized control cultures. As it is typical for whole-culture methods, the degree of synchronization is rapidly lost and is already considerably lower in the second cycle following the block/release protocol.

An aphidicolin block/release protocol was also successfully used to synchronize plant cells of several species, i.e. *Arabidopsis *and tobacco [16,26]. Synchrony was verified by studying the chromosome content using FACS analysis, by quantitation of S-phase cells and by counting metaphase and anaphase cells. An *Arabidopsis *cell line was used to characterize cell cycle-dependent transcript level changes using a microarray that is based on a subgenomic cDNA library, and many transcripts were found to be cell cycle-regulated [16].

Aphidicolin block/relase protocols have also often been used to synchronize human cell lines. However, it has recently been reported for two different cell lines (HL-60, HeLa) that it induces apoptosis preferentially in S-phase cells [27]. Therefore the apparent "synchronization" was caused to a large extend by the selective killing of S-phase cells and the arrest of cells at the entrance of S-phase. The surviving synchronized cells were found to contain DNA damaged by double strand breaks and thus this synchronization procedure is not suitable for human cell lines. In contrast, no decrease in cell viability was observed in *H. salinarum *(Fig. 1), and thus it can be concluded that the method, like in plants, generates highly synchronized cultures. These can yield important information about archaeal cell cycle progression and its regulation.

### Analysis of cell cycle-specific transcriptome changes with DNA microarrays

The application of DNA microarrays for the identification of cell cycle-regulated transcripts has caused considerable discussions. There are two major lines of concern: 1) Independent studies with *S. cerevisiae *and with *S. pombe *all identified a high number of cell cycle-regulated genes, but the overlap was unexpectedly small, e.g. three studies with *S. pombe *identified 407, 747 and 750 cell cycle-regulated genes, but only 171 genes were commonly found in all three studies [12-14]. 2) Using the same set of data, different bioinformatic analysis methods yielded different results, e.g. the original analysis of the results obtained with *C. crescentus *indicated that 553 genes are periodically expressed [20], while the re-analysis of Wichert et al. [28] and of Chen et al. [29] identified only 44 genes and 43 genes, respectively. However, both reanalysis methods relied solely on the periodicity of transcript levels, without taking the degree of regulation into account. Both discussions underscore the great importance of a high-quality method for the analysis of microarray results.

A meta-analysis compared the quality of six different microarray analysis methods [30]. Three non-overlapping benchmarking sets of genes were used, that contained cell cycle genes of yeast, i.e. 1) periodically expressed genes identified in a small-scale study, 2) genes whose promoters were co-immunoprecipitated with known cell cycle transcription factors, and 3) genes that are annotated as "cell cycle genes" in the MIPS database. It was examined how well the six different methods were able to identify genes from these benchmarking sets in the published microarray datasets of the *S. cerevisiae *cell cycle [10,11]. It was revealed that methods that concentrate solely on the periodicity and are independent of the magnitude of expression change do not perform well. Quite unexpectedly it was discovered that visual inspection after bioinformatic removal of unregulated genes performed better than any of the five solely computational methods on two of the benchmark sets. However, at these sets it was done non-blindly and that might have influenced the outcome. On the third set visual inspection was done blindly and the result was comparable to the better computational methods and much better than one computational method [30]. Therefore, on the *S. cerevisiae *cell cycle data set visual inspection is at least comparable to computational methods.

We started to analyze the *H. salinarum *cell cycle data with a commercial DNA microarray analysis software (Genespring, Silicon Genetics) which we have very successfully used in several previous studies [31] (Zaparty et al., in preparation). However, although we applied several different clustering algorithms we were not satisfied with the results. Upon visual inspection it became obvious that they contained false-positives, e.g. transcripts that had an elevated level only at one time point and had a very large standard deviation at this time point. Therefore we decided to use visual inspection for the identification of cell cycle-regulated genes (compare Methods), and found the results superior to the application of a commercial DNA microarray analysis software.

### Comparison of *Halobacterium *with eukaryotes, with *Caulobacter *and with *Sulfolobus*

This study revealed that transcript levels of about 3% of the *H. salinarum *genes are cell cycle-regulated. This fraction is considerably lower than for all other species whose transcriptomes have been studied, i.e. *S. cerevisiae *(about 8%) [30], *S. pombe *(about 10%) [15], *A. thaliana *(about 6%) [16], *Homo sapiens *(about 18% to 28%) [17,19], *C. crescentus *(about 19%) [20], and *S. acidocaldarius *(8.5% or 18%, see below) [21]. In addition to the quantitative difference, there is also a qualitative difference between the transcriptional programs of *H. salinarum *and all other species studied until now. In the four eukaryotes, the transcripts of many important cell cycle proteins were found to be periodically regulated. These include cyclins, checkpoint kinases, regulators and enzymes of nucleotide metabolisms, histones, myosins, kinesins etc.. The conserved core of genes that are cyclic in several species is rather small and thus cell cycle regulation and the level at which it is exerted is poorly conserved in eukaryotes. Even the two yeasts *S. cerevisiae *and *S. pombe *deviate considerably [15,30]. Nevertheless, the regulation of several genes encoding cell cycle proteins is conserved from yeast to humans, including histones, the Cdc6 protein involved in replication initiation, DNA polymerases, and proteins of the SMC family. With only two exceptions, these genes are not cell cycle-regulated in *H. salinarum *(see Additional file 1). This underscores that the conservation of regulatory mechanisms is much lower than the conservation of proteins and their functions.

In *Halobacterium*, only 14 genes with cell cycle-specific functions are regulated on the transcriptional level: one paralog of the *cdc6 *gene (eight paralogs are not regulated), one member of the SMC protein family (three are not regulated), nine genes of the purine biosynthesis pathway (pyrimidine biosynthesis is not regulated) and three additional genes encoding enzymes involved in nucleotide metabolism. This number is not only much smaller than in the four eukaryotes, but also than in the only bacterium investigated, *C. crescentus*. In *Caulobacter*, about 40 genes encoding response regulators, histidine kinases and sigma factors as well as nearly 50 genes encoding proteins involved in DNA replication, nucleotide synthesis, DNA repair, chromosome segregation and cell division were found to be periodic [20].

While this paper was under review the results of a characterization of cell cycle-dependent transcriptome changes of *S. acidocaldarius *were published [21]. 346 transcripts were reported to be differentially regulated, 166 in a "cyclic" way and 200 in a "gradual" way. For two reasons it appears that only part of a cell cycle has been characterized: 1) the transcript profiles of most of the cyclic genes do not relax to their previous level after transient induction and 2) the fraction of genes with known cell cycle-specific functions is identical in the cyclic fraction and the gradual fraction. Therefore it seems that all 346 genes must at least be considered to be cell cycle-regulated. Half of the genes encode hypothetical proteins, a fraction that is considerably higher than in *H. salinarum *(Table 1). Similar to *H. salinarum*, the transcript levels of purine biosynthesis enzymes is regulated, in contrast to the pyrimidine biosynthesis pathway. In total about 30 transcripts encoding proteins with known cell cycle functions are regulated. Encoded proteins include 14 enzymes involved in nucleotide metabolism, three Cdc6 homologs, five additional proteins involved in replication, and four DNA repair proteins.

Taken together, *H. salinarum *has a different regulatory strategy than all other species studied until now. It has a tight cell cycle control, but is lacking periodic transcriptional control of many cell cycle proteins. The data indicate that the control is exerted on the translational and/or posttranslational level. While the characterization of proteome changes is the focus of an independent project, cell cycle-dependent concentration changes of the signaling molecule cAMP could indeed be verified and indicate posttranslational control mechanisms.

### Promoter structure of cell cycle-regulated genes

A shared transcriptional profile of genes indicates that they form a regulon and are regulated by a common transcriptional regulator. Indeed, the comparison of the upstream regions of co-regulated yeast genes allowed the detection of consensus sequences for the binding of known cell cycle-specific transcription factors as well as of novel consensus sequences for unknown factors [11]. Similarly, the comparison of the sequences around the start sites of cell cycle-regulated genes of *H. salinarum *revealed the existence of cluster-specific conserved sequence motifs. Cell cycle transcription factors have not yet been identified for *H. salinarum *or any archaeal species. The cluster-specific conserved motifs will lead to the identification of regulatory proteins and will help to unravel the mechanism of cell cycle-specific transcriptional control in the model archaeon *H. salinarum*.

### The signaling molecule cAMP and its cell cycle-specific concentration changes

It is long known that cAMP has important roles as a signaling molecule in bacteria and in eukaryotes. A very early report revealed that cAMP occurs also in three archaeal species, and it was speculated that it might be a signal for carbon starvation, similar to its role in *E. coli *[33]. But this result was not followed up and the distribution of cAMP in archaea and its functional role remained unknown. A bioinformatics analysis revealed that a gene encoding a putative adenylate cyclase is present in 16 out of the 20 archaeal genomes known in 2004, and that it is absent only in *Picrophilus torridus, Nanoarchaeum equitans*, and two species of *Thermoplasma *[34].

Here we show that *H. salinarum *contains on average about 1 – 2 × 10^5 ^cAMP molecules per cell. The amount is in congruence with the number of about 5 × 10^6 ^ATP molecules per cell and shows that several percent of ATP are converted to cAMP. The genome of *H. salinarum *contains a gene for the enzyme that catalyzes this conversion, i.e. an adenylate cyclase (OE2856F). A blast search led to the detection of 28 archaeal orthologs and underscored that this enzyme is widespread in archaea. Therefore it can be concluded that cAMP plays a role as a signaling molecule in most archaeal species. A gene encoding a phosphodiesterase could not be identified in the genome of *H. salinarum *by similarity searches using sequences of known cAMP phosphodiesterases, and thus the identity of the enzyme for cAMP degradation cannot be predicted.

If a typical *H. salinarum *cell is approximated by a cylinder of 4 μm length and 0.5 μm diameter, the amount of 1 – 2 × 10^5 ^cAMP molecules per cell are equivalent to an intracellular concentration of about 200 μM. This is considerably higher than reported for *S. cerevisiae*. The volume of a typical yeast cell is about 100 fl. Therefore the reported cAMP values are equivalent to concentrations of 0.3 μM, 1 μM and 6 μM. The cAMP level in synchronized yeast cultures was measured in three independent studies, and they all agree that there are cell cycle-specific fluctuations [35-37]. Müller et al. [35] reported sharp increases in the cAMP concentration that occur at different times in the cell cycle depending on glucose availability. They point out that cAMP integrates energy metabolism and cell cycle control in yeast. Another eukaryote, *Tetrahymena pyriformis*, was found to have one short peak of elevated cAMP concentration precisely at the time of cell division [38]. In *H. salinarum *two short cAMP peaks were found prior to and after cell division, while the concentration dropped to average level in between. This pattern is different from the concentration changes reported for eukaryotic species. However, the common theme in all species is a very short time of concentration change.

It remains to be discovered whether the cAMP peaks in *H. salinarum *are instrumental for cell cycle progression, and whether cAMP is also involved in metabolic control. In conclusion, it has been revealed for the first time that the cAMP level is sharply and reversibly elevated at two points during an archaeal cell cycle and may thus play a similar role than proposed for several eukaryotic species. If cAMP oscillations could be detected in further archaeal and eukaryotic species, this would indicate an early evolutionary role of cAMP in cell cycle signaling. In addition, it might be an evolutionary old signal for the integration of the energy status and cell cycle progression.

## Conclusion

*H. salinarum *is one of two archaeal and of very few prokaryotic species that can be synchronized. The degree of synchrony is very high and thus *H. salinarum *is ideally suited to study cell cycle-dependent processes. In this and the previous study [22] it was used to characterize various cell cycle-dependent processes, i.e. 1) septum formation and cell division, 2) specific intracellular DNA localization and its dynamics, 3) transcripts level changes of selected genes, 4) changes of the transcriptome, 5) Sph1 protein level oscillations, and 6) concentration changes of the signaling molecule cAMP.

**Figure 8:**
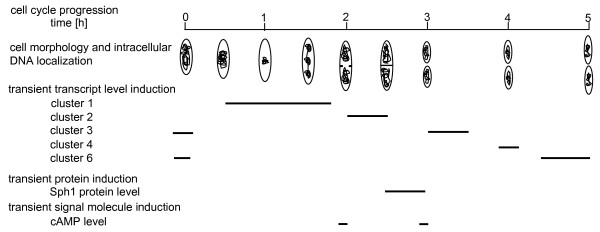
**Overview of characterized cell cycle-dependent processes in *H. salinarum***. The Figure summarizes schematically the results obtained in this study and previously published results [22].

Fig. 8 gives an overview of the different events. At all levels (transcript, protein, small molecules, intracellular transport) tight cell cycle-dependent regulation was observed. The reversible inductions follow a strict time schedule, which is especially evident for the fast oscillations of the cAMP concentration. The virtual absence of transcriptional regulation of genes encoding regulatory proteins indicates that a major part of cell cycle regulation probably occurs at the posttranscriptional level. The existence of at least one cell cycle checkpoint in *H. salinarum *can be concluded, because cell division and several additional cell cycle events are inhibited after a block in replication, and are executed synchronously after release of the block. Further studies will identify changes of the proteome and the role of cell cycle proteins for cell cycle progression.

## Methods

### Strains and culture conditions

The *Halobacterium salinarum *type strain was obtained from the German Culture Collection ("Deutsche Sammlung für Mikroorganismen und Zellkulturen", Braunschweig, Germany; Strain No. DSM670). [39] It was grown in complex medium as described [40].

### Synchronization of *H. salinarum *cultures

Each experiment was started with a single colony that was freshly grown on solid complex medium. It was used to inoculate 20 ml complex medium in a 100 ml Erlenmeyer flask. After two days of incubation at 42°C in a rotary shaker (250 rpm), it was checked that the resulting culture was in mid-exponential growth phase (2 – 6 × 10^8 ^cells/ml). An aliquot was used to inoculate a further culture, which was verified to be in mid-exponential growth phase one day later. The required inoculum was calculated using a generation time of 4 hours. A third culture was inoculated from the second culture. The inoculum was calculated so that the third culture had a cell density of 2 – 4 × 10^8 ^cells/ml the following morning. In this way it was guaranteed that the culture had been growing in exponential growth phase for more than 40 generations.

To start the synchronization, aphidicolin was added to a final concentration of about 30 μM. Aphidicolin was purchased from Serva (Heidelberg, Germany) and dissolved at a concentration of 30 mM in DMSO. Aliquots of this solution were stored at -80°C and only thawed once. The exact concentration to reach an ideal synchronization result was readjusted empirically for every new lot of aphidicolin (concentration range of 25 – 35 μM). Aliquots were withdrawn from the aphidicolin-containing culture and used to determine the cell density (using a Neubauer counting chamber) and the average cell length. The average cell length and its standard deviation were calculated from the lengths of 50 cells that had been determined using an ocular micrometer. The culture was incubated until the average cell length reached 5.7 μm. The cells were harvested by centrifugation (4500 g, 6 min., 42°C, pre-warmed rotor and centrifuge chamber). The pellet was resuspended in pre-warmed medium and the cells were incubated for 10 minutes in the rotary shaker to wash away residual aphidicolin. The cells were washed again as described above. Progression of the resulting synchronized culture through the cell cycle was followed by determination of the cell density and the average cell length at regular intervals. Synchronized cultures were used to determine the cell cycle dependent alterations of the transcriptome, selected transcript levels, and the cAMP level, as described in the Results section.

### Transcriptome analysis with a DNA microarray

For the analysis of the transcriptome throughout the cell cycle, every culture of the three biological replicates was synchronized as described above, and a control culture was treated identically, except that the addition of aphidicolin was omitted. Aliquots of 9 × 10^8 ^cells were removed from both cultures at ten time points. RNA was isolated with the RNA isolation mini kit (Qiagen, Hilden, Germany) according to the manufacturer's instructions, but omitting the incubation with lysozyme. A DNA contamination was excluded by a DNase treatment while the RNA was bound to the ion exchange column. The RNA concentration was determined photometrically, and the integrity was checked by analytical agarose gel electrophoresis. The control RNA samples were pooled, thus generating an unsynchronized control averaged over the 5 hour period that was chosen to study the synchronized culture. Notably the control had experienced the same handling steps (centrifugation, resuspension) as the synchronized culture.

cDNA synthesis from sample and control RNA (15 μg each), concomitant labeling with Cy3-dUTP or Cy5-dUTP, mixing of sample and control, removal of unincorporated label, and concentration were performed as described previously [31].

The DNA microarray for *H. salinarum *was constructed in collaboration with the group of Dieter Oesterhelt (MPI for Biochemistry, Martinsried). It contains gene-specific PCR products for more than 95% of the nearly 2800 genes that were annotated within the genome sequencing project of the Oesterhelt group in April 2002 [23]. Construction of the DNA microarray will be described in detail elsewhere (Lange et al., in preparation). Slide preparation, competitive hybridization of samples from synchronized cultures and the pooled unsynchronized control, washing and drying of slides were performed as described previously [31], except that a hybridization temperature of 67°C was used. The slides were scanned using an Axon 4000A scanner (Axon Instruments, Union City, CA, USA), laser intensities were chosen to get roughly equal average signals for the Cy3- and Cy5-channel.

### Analysis of DNA microarray data

Scanning of 30 DNA microarrays (10 cell cycle time points, triplicate measurements), each containing about 2800 gene-specific spots, had generated more than 80000 data points. After automatic spot detection and signal integration, all data points were visually inspected using the software Genepix Pro 3.0 (Axon Instruments, Union City, CA, USA). Weak spots and spots containing fluorescence artifacts were removed from the analysis, and, if necessary, the software-generated area used for signal detection was manually adjusted. For each microarray a table containing the results as tab-delimited text was exported from Genepix Pro 3.0 and imported into Excel (Microsoft, Redmond, USA). The following criteria were used to filter and normalize the data: 1) all data points were removed that had a signal to background ratio of less than three, 2) all data points that after background subtraction had lower fluorescence intensities than 1000 a.u. were removed, 3) the average of all Cy-3 signals and all Cy-5 signals were normalized to equity assuming that the majority of genes are not cell cycle-regulated. Determination of cell density, average cell length and fraction of dividing cells had revealed that the time window of cell division in one of the three experiments had an offset of 30 minutes in comparison to the other two experiments. While the reason for the difference is not known, the observed offset was corrected by "re-timing" this data set. The consequences are 1) that the five hour time point is represented only by two values instead of three and 2) that an eleventh time point without replicates was generated at 30 minutes, when no culture aliquot had been removed.

After that, all 30 results tables were loaded into the DNA microarray analysis software Genespring (Silicon Genetics, Redwood City, CA, USA). The definition file for the *H. salinarum *DNA microarray was also loaded, and a time course experiment with triplicate measurements was set up. All genes were removed with signals for less than 7 out of the 11 time points, leaving a data set of 2457 genes. Next, all genes were removed from the results set that at none of the 11 time points deviated from average by more than 30% (all Cy3 to Cy5 signal intensity ratios between 0.7 and 1.3). This excluded 661 genes that with great certainty were not cell cycle-regulated and had an average transcript level even at the time point without replicates. 1796 genes were left that had the potential to be cell cycle-regulated. However, most of them had a Cy3/Cy5-signal ratio of smaller than 0.7 or of greater than 1.3 at a single time point. Typically, these values had a high variance, and thus these genes were not regarded to be cell cycle-regulated. To identify cell cycle-regulated genes, the transcript level profiles of all 1796 genes were visually inspected with Genespring, and cell cycle-regulated genes were identified. Criteria for identification were up- or downregulation at two or more subsequent time points and a ratio of highest to lowest level of at least two. The inspection was done "blindly", meaning that gene name and function were not known as the transcription profiles were examined. 87 cell cycle-regulated genes were discovered. Most of them showed transcription profiles that could be grouped into one of seven clusters (compare Results).

### Northern blot analysis

Northern blot analysis was performed as described [22]. In short, RNA was isolated using the method of Chomczynski and Sacchi [41]. It was size-fractionated on formaldehyde agarose gels, transferred to nylon membranes by downward capillary blotting [42] and immobilized by UV crosslinking. Digoxigenin-labelled probes were constructed as described [22] using the oligonucleotides summarized in Additional file 2. Hybridization was performed under standard stringency conditions (50% formamide, hybridization temperatures see see Additional file 2). The membranes were washed successively in 2 × SSC, 1 × SSC, 0.5 × SSC and 0.25 × SSC. All buffers contained 0.1% SDS. The first wash step were performed at room temperature, the last three steps at 5°C below the hybridization temperature. DIG detection was performed with an enzyme linked anti-DIG antibody and the chemiluminescense substrate CDP-star according to the manufacturer's instruction (Roche, Mannheim, Germany). The signals were visualized with X-ray films. All the probes were specific and led to the detection of a single band. Transcript sizes were determined with an RNA standard (MBI Fermentas, St. Leon-Rot, Germany) and were found to be in excellent agreement with the sizes of monocistronic transcripts of the genes (OE5212F, OE4676F, OE1679R, OE5211F, OE4380F, OE3571F, OE1414R, OE5049F) or with polycistronic transcripts of two or more adjacent genes (OE1797R/OE1794R, OE4418R/OE4419R/OE4420R; OE4300R/OE4301R/OE4302R/OE4304R, OE2019F/OE2020F, OE4345R/OE4346R, OE3571R/OE3572R).

### Identification of cluster-specific conserved sequence motifs

The sequences from -200 nt to +200 nt around the translational start site were retrieved from the genome sequence [23] for all genes that were presumed to have a cell cycle-regulated promoter. If the intergenic region of two genes was smaller than 40 nt, they were treated as a putative operon and only the first gene was included in the analysis. Cluster six contains only two genes in a putative operon and thus could not be analyzed. The genes of the remaining six clusters were searched for cluster-specific conserved motifs using several programs, i.e. "Structure Logo", "ClustalW", "AlignAce", and "MEME". Structure Logo and ClustalW allowed to retrieve the TATA box and BRE, indicating that most transcripts are leaderless and translational and transcriptional start points (nearly) coincide. However, they did not lead to the detection of cluster-specific conserved motifs, indicating that regulatory elements, if they exist, do not have a fixed distance to the translational/transcriptional start point. The analyses with AlignAce [43] and MEME (Multiple EM for Motif Elicitations) [44,45] were performed using two websites [46,47]. Both programs led to the detection of conserved motifs for all six clusters. The putative regulatory motifs indeed have variable distances to the translational/transcriptional start points. The results of MEME are summarized in Table 2.

### Determination of cAMP levels

Aliquots containing 1 × 10^9 ^cells were removed from synchronized cultures at the times indicated in the Results section. The cells were harvested by centrifugation (18 000 g, 1 min., room temperature). The pellet was quickly resuspended in 900 μl of cold double-distilled water (a. bid.), resulting in total lysis of the cells by osmotic shock. 100 μl of 1 N HCl was added and it was mixed thoroughly. Care was taken that cell lysis and pH downshift were performed in less than 30 seconds. The suspension was centrifuged (18 000 g, 5 min., 4°C), the supernatant was quickly frozen in liquid nitrogen and stored at -80°C.

For cAMP determination, the samples were thawed on ice. 100 μl aliquots were used to quantitate the cAMP concentration with the "Direct cAMP Enzyme Immunoassay Kit" (Assay Designs Inc., Ann Arbor, MI, USA) according to the instructions of the manufacturer. A standard cAMP curve and several controls were included in the kit. All samples were determined in duplicates.

### Database submission

The microarray results have been submitted to the database "ArrayExpress" [48] and got the accession No. E-MEXP-1033. The experiment has the name "JWGU-Soppa-Halobacterium-salinarum-cell-cycle-regulation".

## Authors' contributions

AB performed the experimental work and constructed Figures and Tables, CL produced the DNA microarrays and helped in the analysis of DNA microarray experiments, JS discussed experimental strategies and wrote the manuscript. All authors have read and approved the final manuscript.

## Supplementary Material

Additional file 1 Transcript level profiles of selected unregulated genes. The reasons for gene selection are explained in the text. The transcript level profiles of genes encoding the following proteins are shown: 1) all enzymes for pyrimidine biosynthesis, 2) all members of the SMC protein family, 3) all Cdc6 paralogs that are represented on the microarray, 4) selected cell cycle proteins that have periodic transcripts in several eukaryotes, and 5) homologues of the bacterial cell division protein FtsZ. Click here for file

Additional file 2 Oligonucleotides used for Northern blot analysis. The Table contains the names of all oligonucleotides, their sequence, the name of the target gene, length of the probe generated by PCR, and hybridization temperature. Click here for file
