# Supplementary figures and images for: Transcriptome changes and cAMP oscillations in an archaeal cell cycle
Source: BMC Cell Biol. 2007 Jun 11;8:21. doi: 10.1186/1471-2121-8-21 (PMC1906763; doi:10.1186/1471-2121-8-21)

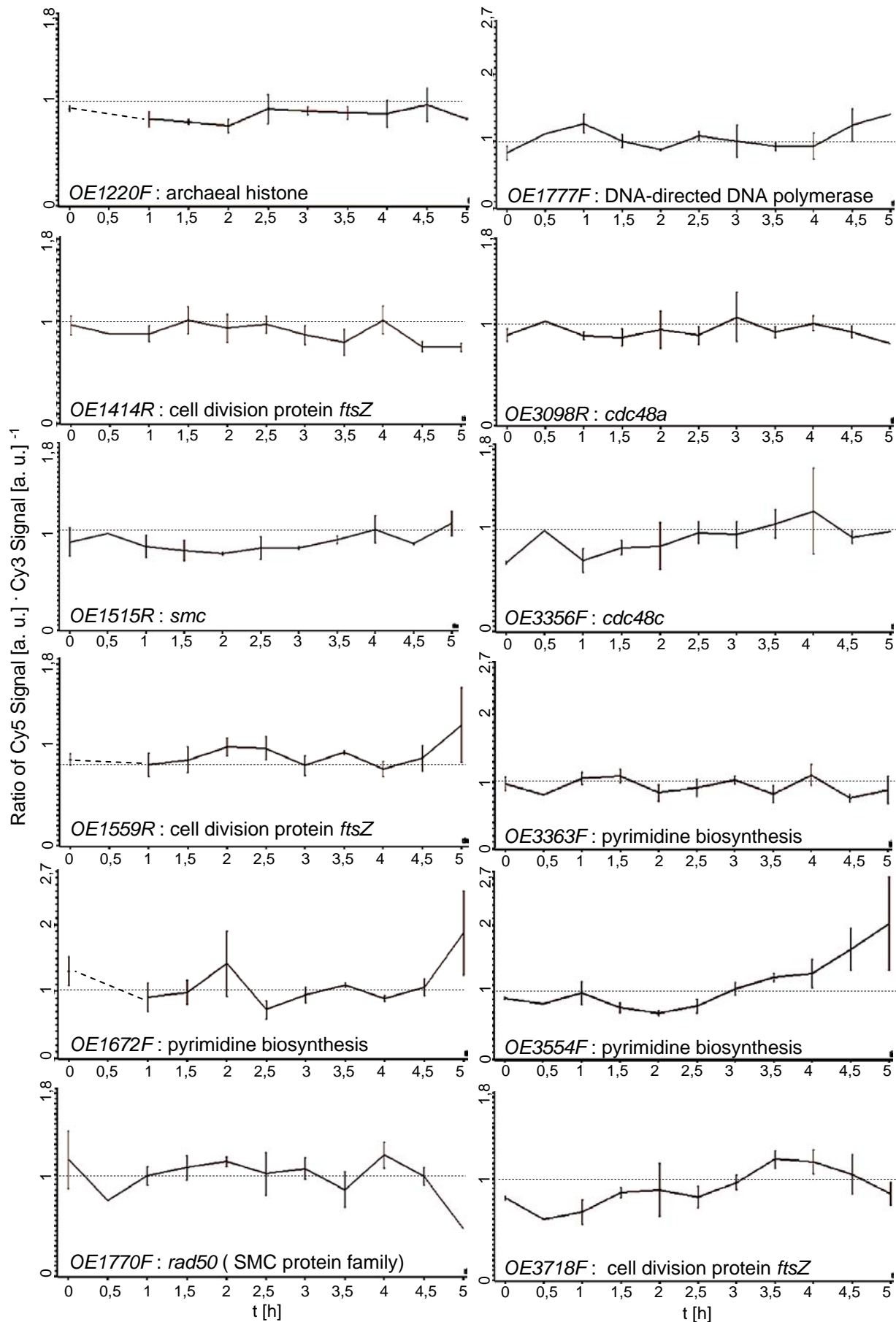

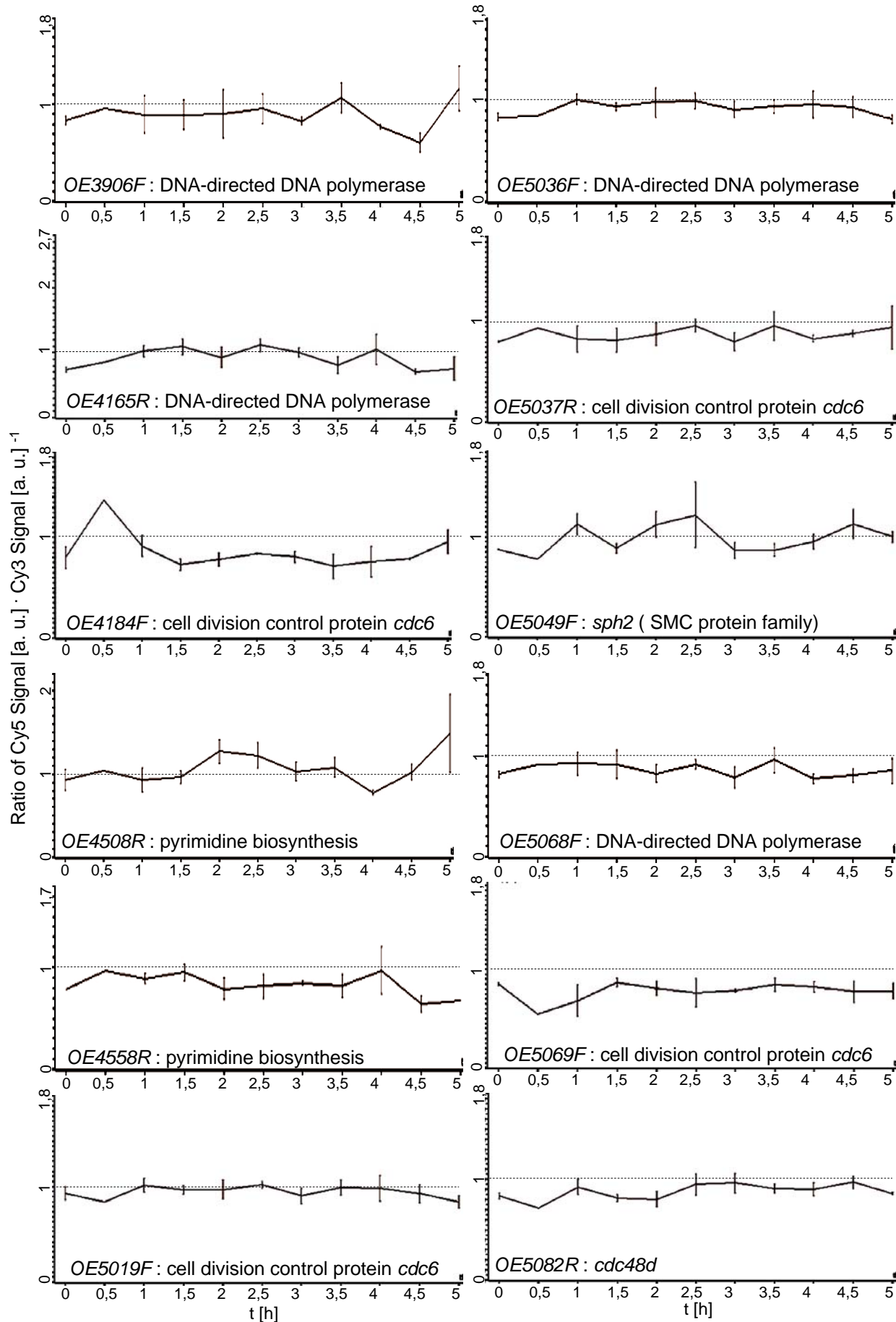

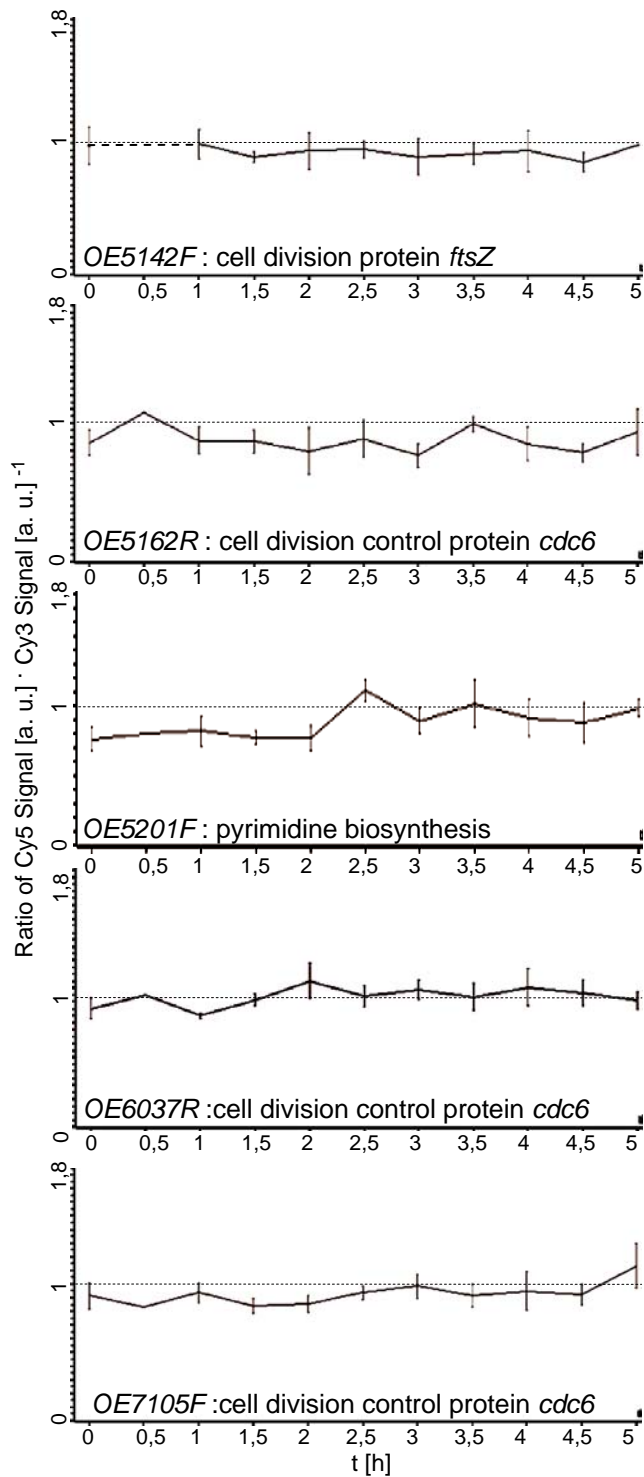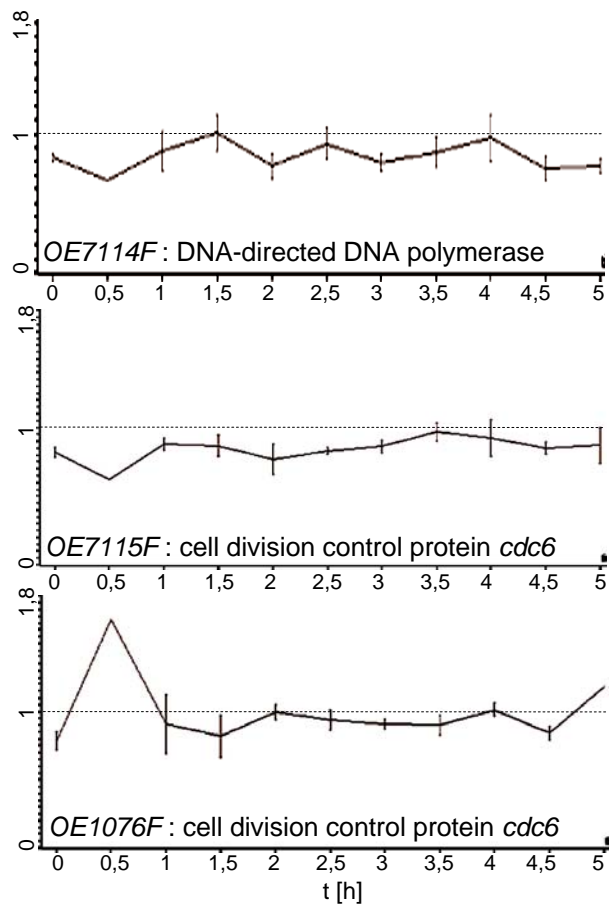

Supplement: Additional file 1 — Transcript level profiles of selected unregulated genes. The reasons for gene selection are explained in the text. The transcript level profiles of genes encoding the following proteins are shown: 1) all enzymes for pyrimidine biosynthesis, 2) all members of the SMC protein family, 3) all Cdc6 paralogs that are represented on the microarray, 4) selected cell cycle proteins that have periodic transcripts in several eukaryotes, and 5) homologues of the bacterial cell division protein FtsZ. [file 1471-2121-8-21-S1.pdf]
