# Supplementary material for: Transcriptome changes and cAMP oscillations in an archaeal cell cycle
Source: BMC Cell Biol. 2007 Jun 11;8:21. doi: 10.1186/1471-2121-8-21 (PMC1906763; doi:10.1186/1471-2121-8-21)
Supplement: Additional file 2 — Oligonucleotides used for Northern blot analysis. The Table contains the names of all oligonucleotides, their sequence, the name of the target gene, length of the probe generated by PCR, and hybridization temperature. [file 1471-2121-8-21-S2.pdf]

primer name	sequence	target gene	length of probe	hybridization temperature
OE4345R_Sonde1F	GAAGAGGTGGCGATGCAAGG	ribonucleoside-diphosphate reductase (EC 1.17.4.1) beta chain	398bp	50°C
OE4345R_Sonde1R	ACGAGATCCCGCAGGAAGTC			
OE4304R_Sonde1F	CGTGCGATGCCGAACTTCTG	ABC-type transport system permease protein	448bp	55°C
OE4304R_Sonde1R	AGCCGCCGATGAGCAAGTAG			
OE2019F_Sonde1F	AGTCGAGATGGCCGAGGAGTTC	fructose-bisphosphate aldolase (EC 4.1.2.13) 1	300bp	50°C
OE2019F_Sonde1R	CGATTGCGCCCTTCACGGATTC			
OE3571R_Sonde1F	TCGTGCAGGGCACCATCTAC	GMP synthase (glutamine-hydrolyzing) (EC 6.3.5.2), subunit B	260bp	50°C
OE3571R_Sonde1R	ACAGACCGCGACCTTCTCAG			
OE1414R_Sonde1F	TGACGAGGGCGGCATCTATC	cell division protein *ftsZ4*	373bp	50°C
OE1414R_Sonde1R	GGTCTGTGGCACGCTCATTC			
OE4419Sonde1F	GCCGTGATTTCCTGCTGGAG	argininosuccinate lyase (EC 4.3.2.1)	164bp	45°C
OE4419Sonde1R	GTCCGGGTTCTTCTTCTGGG			
OE4676 HP F Sonde_1	GCACAGACTTCGGACGACTC	hypothetical protein	150bp	45°C
OE4676 HP R Sonde_1	GCCACAGACTGTGCAGGTTC			
SIR R Sonde_1 F	TGGCGACCCATCTGGACTAC	transcription regulator *sirR*	310bp	45°C
SIR R Sonde_1R	TGCTCGGTGACCGTGAGTTC			
ORC 7 Sonde_1F	AAGACGGGCACCGGAAAGAC	cell division control protein *cdc6* homolog	310bp	45°C
ORC 7 Sonde_1R	CGAAACATCGGAGCCCTCAC			
OE 1679 Sonde_1 F	GGTTCGCCAACCACTTCTGC	probable ABC-type phosphate transport system periplasmic phosphate-binding protein	400bp	45°C
OE 1679 Sonde_1 R	TGGATGATGGTCCGGTCCTG			
hp24 F Sonde1	GACGGACCACTGCTGTAAG	conserved hypothetical protein	800bp	50°C
hp24 R Sonde1	CTCGACAGATGAACCCACG			
Sph1 F Sonde1	GCTTCGCGTTGTTCGATC	SMC-like protein *sph1*	1050bp	55°C
Sph1 R Sonde1	TGGAGAACGAAGACGACGG			
Sph2 Sonde1 F	CAGAGCGTCGTGACTTGG	SMC-like protein *sph2*	716bp	50°C
Sph2 Sonde1 R	GCTGTGATTCACGCAGTTCC			
